# Supplementary material for: Content-rich biological network constructed by mining PubMed abstracts
Source: BMC Bioinformatics. 2004 Oct 8;5:147. doi: 10.1186/1471-2105-5-147 (PMC528731; doi:10.1186/1471-2105-5-147)
Supplement: Additional File 2 — The original results of the above study (non-essential files are deleted to keep the file size under the limit set by BMC bioinformatics). [file 1471-2105-5-147-S2.bz2 › chilibotAdditionalFile2/dip40/22ID9129204E80/html/FADD_TNFRSF1A.html]

 


 **FADD** and **TNFRSF1A** 
  
Found 377 abstracts in PubMed, retrieved 40.  
 

 What does Google say? 
 PDF only 
| .edu only 

---

**Interactive relationship** (e.g. stimulation, inhibition, etc)

**Inhibitory relationship**- Neutralizing anti TNF R1  [ **TNFRSF1A** ]  antibodies inhibited macrophage activation assessed by Fas  [ **FADD** ]  L expression and NO secretion.  Ref: 12869351 Arterioscler Thromb Vasc BiolArterioscler Thromb Vasc Biol,
- Our results show, that the exposure of three metastatic melanoma cell lines A2058, SK Mel 5, and WM 266 4 to recombinant TIMP 3, N terminal MMP inhibitory domain of TIMP 3, as well as to adenovirally expressed TIMP 3 results in stabilization of tumor necrosis factor receptor 1  [ **TNFRSF1A** ]  TNF RI  [ **TNFRSF1A** ] , FAS  [ **FADD** ] , and TNF related apoptosis inducing ligand receptor 1 TRAIL RI on melanoma cell surface and sensitizes these cells to apoptosis induced by TNF alpha, anti Fas  [ **FADD** ]  antibody and TRAIL.  Ref: 12687014 Oncogene, 2003
**Stimulatory relationship**- In addition, NF kappaB downstream of TNFR  [ **TNFRSF1A** ]  was required for upregulation of Fas  [ **FADD** ]  expression by endogenous TNF secreted in response to TCR stimulation.  Ref: 12813034 J Biol Chem< MedlineTA>J Biol Chem, 2003
- During endotoxin induced systemic inflammation, both TNFR  [ **TNFRSF1A** ]  p55 and TNFR  [ **TNFRSF1A** ]  p75 appear to be of minimal importance for modulation of Fas  [ **FADD** ]  mediated apoptosis and associated A1 protein expression despite normal Fas  [ **FADD** ]  TNFR  [ **TNFRSF1A** ]  p55 and increased TNFR  [ **TNFRSF1A** ]  p75 expression in neutrophils.  Ref: 12630518 Shock, 2003
- Expression of TNFR1  [ **TNFRSF1A** ]  gene increased 2.7 fold and expression of Fas  [ **FADD** ]  gene decreased 3.1 fold compared to intact endosalpinx, which indicates induction of apoptosis triggered by tumor necrosis factor alpha and inhibition of the Fas  [ **FADD** ]  dependent pathway.  Ref: 12802421 Bull Exp Biol Med< MedlineTA>Bull Exp Biol Med, 2003
**Neutral relationship**- Apoptosis induced by TNF receptor I TNFR1  [ **TNFRSF1A** ]  is thought to proceed via recruitment of the adaptor  **FADD**  and caspase 8 to the receptor complex.  Ref: 12887920 Cell, 2003
- Fas  [ **FADD** ]  is a death receptor belonging to the TNFR  [ **TNFRSF1A** ]  superfamily and induces cell apoptosis by both activating a caspase cascade and altering mitochondria.  Ref: 12697265 Life Sci, 2003
- Fas  [ **FADD** ]  CD95 or APO 1 is a member of tumor necrosis factor receptor TNFR  [ **TNFRSF1A** ]  family.  Ref: 12806430 Drug News Perspect, 2001
- The Fas  [ **FADD** ]  associated death domain  **FADD**  adaptor protein  **FADD**  Mort 1  [ **FADD** ]  is recruited by several members of the tumor necrosis factor receptor TNFR  [ **TNFRSF1A** ]  superfamily during cell death activated via death receptors.  Ref: 12815462 Cell Death Differ, 2003

**Non-interactive relationship** (e.g. studied together, co-existance, homology, etc.)

- Fas  [ **FADD** ]  CD95, APO 1 is regarded as the prototypical cell death receptor of the TNFR  [ **TNFRSF1A** ]  superfamily.  Ref: 12794152 J Immunol, 2003
- METHODS Expressions of T cell apoptosis promoting and inhibiting genes, including Fas  [ **FADD** ] , FasL, Bcl 2, Bax, TNFR1  [ **TNFRSF1A** ]  and TNFR2, as well as activity of cysteine proteinase in cascade connection, such as Caspase 8 and Caspase 3 were determined by TUNEL labeled flow cytometry and fluorescence real time quantitative RT PCR technique.  Ref: 12585108 Zhongguo Zhong Xi Yi Jie He Za Zhi, 2002
- The kinetics of apoptosis and the expression of Fas  [ **FADD** ]  and TNF R1  [ **TNFRSF1A** ]  were also assessed.  Ref: 12615653 Brain, 2003
- As several pathways are involved in induction of apoptosis of T cells, including transforming growth factor TGF beta TGF receptor TGFR, TNF alpha TNFR1  [ **TNFRSF1A** ] , and Fas  [ **FADD** ]  Fas  [ **FADD** ]  ligand, these mediators were also investigated in peripheral blood samples from these subject groups.  Ref: 12851215 Am J Physiol Lung Cell Mol Physiol< MedlineTA>Am J Physiol Lung Cell Mol Physiol, 2003
- Noggin overexpression leads to reduction of apoptosis and retardation of cell differentiation in the eyelid epithelium, which is associated with downregulation of expression of the apoptotic receptors Fas  [ **FADD** ] , p55 kDa TNFR  [ **TNFRSF1A** ] , Id3 protein and keratinocyte differentiation markers loricrin, involucrin.  Ref: 12805214 EMBO J< MedlineTA>EMBO J, 2003
- In an in vitro binding assay, the intracellular domain of TNF R1  [ **TNFRSF1A** ]  bound TRADD, RIP and TRAF2 but did not bind  **FADD**  or caspase 8.  Ref: 12721308 J Biol Chem< MedlineTA>J Biol Chem, 2003
- Finally, intestinal adaptation and apoptosis were recorded in the remnant ileum after SBR in TNFR1  [ **TNFRSF1A** ]  null and Fas  [ **FADD** ]  null mice.  Ref: 12724132 Am J Physiol Gastrointest Liver Physiol< MedlineTA>Am J Physiol Gastrointest Liver Physiol, 2003
- This includes radiation induced signaling via the EGFR and IGFI R to the PI3K, MAPK, JNK, and p38 pathways as well as FAS  [ **FADD** ]  R and TNF R  [ **TNFRSF1A** ]  signaling to pro caspases and NFKB.  Ref: 12600231 Radiat Res, 2003
- Because cellular FLICE caspase 8 inhibitory protein cFLIP is a recently identified intracellular inhibitor of caspase 8 activation that potently inhibits death signaling mediated by all known death receptors, including Fas  [ **FADD** ] , TNF receptor TNF R  [ **TNFRSF1A** ] , and TNF related apoptosis inducing ligand receptors TRAIL Rs, we investigated the expression and function of cFLIP in human HCCs.  Ref: 12861043 Lab Invest, 2003
- The purpose of this study was to elucidate the role s of TNFR  [ **TNFRSF1A** ]  p55 and TNFR  [ **TNFRSF1A** ]  p75 in Fas  [ **FADD** ]  mediated neutrophil apoptosis and A1 expression in a mouse model of endotoxemia.  Ref: 12630518 Shock, 2003
- Adenovirus mediated expression of TIMP 3 in human melanoma xenografts in vivo resulted in increased immunostaining for TNF RI  [ **TNFRSF1A** ] , FAS  [ **FADD** ] , and cleaved caspase 3, and in apoptosis of melanoma cells.  Ref: 12687014 Oncogene, 2003
- Thus, p75 NTR may function as a death receptor similar to Fas  [ **FADD** ]  or TNFR  [ **TNFRSF1A** ]  I or a dependence receptor similar to deleted in colorectal cancer DCC or uncoordinated gene 5 homologues 1 3 UNC5H1 3.  Ref: 12787561 Cytokine Growth Factor Rev, 2003
- In the setting of accelerated apoptosis, TNFR1  [ **TNFRSF1A** ]  and Fas  [ **FADD** ]  expression were paradoxically decreased.  Ref: 12724132 Am J Physiol Gastrointest Liver Physiol< MedlineTA>Am J Physiol Gastrointest Liver Physiol, 2003
- Measles virus induced Fas  [ **FADD** ]  but not FasL or TNFR1  [ **TNFRSF1A** ]  expression on PMBC, as well as activation of granzyme B and caspase cascade.  Ref: 12713458 Eur J Clin Invest, 2003
- Membrane associated receptors Fas  [ **FADD** ] , TNFR  [ **TNFRSF1A** ]  p55 and TNFR  [ **TNFRSF1A** ]  p75 and cytoplasmic A1 expression of freshly isolated neutrophils were assessed by one color flow cytometry and western blotting respectively.  Ref: 12630518 Shock, 2003
- The fusion of the transmembrane domain of LMP1 to signaling domains of CD40, TNF R1  [ **TNFRSF1A** ]  and Fas  [ **FADD** ]  activates their signaling.  Ref: 12805217 EMBO J, 2003
- on the other hand, its death domain is of type II unlike that of Fas  [ **FADD** ]  or TNFR  [ **TNFRSF1A** ]  I, and it has also been shown to be capable of mediating cell death in response to the withdrawal of ligand.  Ref: 12787561 Cytokine Growth Factor Rev, 2003
- However, unlike most  **FADD**  dependent apoptosis, this pathway may not involve death receptors, including Fas  [ **FADD** ] , tumor necrosis factor receptor 1  [ **TNFRSF1A** ] , or tumor necrosis factor related apoptosis inducing ligand receptors, because inhibitors of the receptors did not prevent DT GMCSF induced apoptosis.  Ref: 12576460 Clin Cancer Res, 2003
- cFLIP down regulation induced by cFLIP antisense oligodeoxynucleotides sensitized HLE cells to Fas  [ **FADD** ] , TNF R  [ **TNFRSF1A** ] , and TRAIL R mediated apoptosis.  Ref: 12861043 Lab Invest, 2003
- Ileal TNFR1  [ **TNFRSF1A** ]  and Fas  [ **FADD** ]  expression were then measured after SBR in the setting of increased waved 2 mice or decreased exogenous EGF administration apoptosis.  Ref: 12724132 Am J Physiol Gastrointest Liver Physiol< MedlineTA>Am J Physiol Gastrointest Liver Physiol, 2003
- The chicken death receptors, Fas  [ **FADD** ] , TNFR1  [ **TNFRSF1A** ] , DR6, and TVB, are constitutively expressed in a relatively wide variety of hen tissues.  Ref: 12878204 Biochem Biophys Res Commun, 2003
- Apoptotic and adaptive responses were preserved in both TNFR1  [ **TNFRSF1A** ]  null and Fas  [ **FADD** ]  null mice.  Ref: 12724132 Am J Physiol Gastrointest Liver Physiol< MedlineTA>Am J Physiol Gastrointest Liver Physiol, 2003
- TNFR 1  [ **TNFRSF1A** ]  p55 and Fas  [ **FADD** ]  share a death domain which is critical for apoptosis signaling whereas TNFR  [ **TNFRSF1A** ]  p55 and TNFR  [ **TNFRSF1A** ]  2 p75 can activate NF kappaB leading to anti apoptotic proteins expression such as A1.  Ref: 12630518 Shock, 2003
- Eight human DRs Fas  [ **FADD** ] , TNF R1  [ **TNFRSF1A** ] , TRAMP, TRAIL R1, TRAIL R2, DR 6, EDA R and NGF R have been identified.  Ref: 12655300 Cell Death Differ, 2003
- CONCLUSIONS IL 6 in contrast to its anti apoptotic modulation of the Fas  [ **FADD** ]  induced pathway exerted a pro apoptotic effect on the TNFalpha actinomycin D induced apoptosis by increasing the number of TNF R  [ **TNFRSF1A** ]  on hepatocytes.  Ref: 12763364 J Hepatol, 2003
- We first performed SBR or sham operations in mice and the temporal expression of caspases, and 3, death receptors TNFR1  [ **TNFRSF1A** ]  and Fas  [ **FADD** ] , and corresponding ligands TNF and Fas  [ **FADD** ]  L were determined in the remnant intestine at various postoperative time points.  Ref: 12724132 Am J Physiol Gastrointest Liver Physiol< MedlineTA>Am J Physiol Gastrointest Liver Physiol, 2003
- Based on the structural and functional differences between p75NTR and the related receptors Fas  [ **FADD** ]  or TNFR  [ **TNFRSF1A** ] , it has been suggested that these receptors have distinct signaling functions.  Ref: 12852261 Vitam Horm, 2003
- Death receptors include tumor necrosis factor receptor 1  [ **TNFRSF1A** ] , Fas  [ **FADD** ] , death receptor DR 3, DR4, DR5 and DR6.  Ref: 12604411 Haematologica, 2003
- Significantly increased apoptosis of PHA stimulated T cells was observed in COPD annexin positive 75.0 14.7% SD vs. control 50.2 21.8% SD, P = 0.006, along with upregulation of TNF alpha TNFR1  [ **TNFRSF1A** ] , Fas  [ **FADD** ] , and TGFR.  Ref: 12851215 Am J Physiol Lung Cell Mol Physiol< MedlineTA>Am J Physiol Lung Cell Mol Physiol, 2003
- In the present study, we investigated whether the expression of the TNFR  [ **TNFRSF1A** ]  family death domain receptor, Fas  [ **FADD** ] , and its ligand, FasL, is altered in association with neuropathology and activated caspase markers in Alzheimer disease AD brain, and Abeta induced neuronal cell death in vitro.  Ref: 12742739 Neurobiol Dis, 2003
- We tested the hypothesis that activation of specific apoptotic pathways represents a mechanism for tubular cell death after CAD kidney transplantation.METHODS Serial tissue sections from paraffin embedded needle biopsy specimens obtained at approximately 1 hr of reperfusion after transplantation of 13 CAD and 12 living related donor LRD renal allografts were examined by using the terminal deoxynucleotide transferase mediated dUTP nick end labeling assay to detect apoptosis and by immunohistochemistry for expression of key pro apoptotic molecules Bax, Bak, tumor necrosis factor receptor TNFR 1  [ **TNFRSF1A** ] , Fas  [ **FADD** ] , and cytochrome c.  Ref: 12865785 Transplantation, 2003
- Fas  [ **FADD** ]  and TNFR  [ **TNFRSF1A** ]  p55 expression was normal and TNFR  [ **TNFRSF1A** ]  p75 was significantly increased in all the LPS injected WT and KO mice although absence of the appropriate surface receptors was confirmed in the KO mice.  Ref: 12630518 Shock, 2003
- While recruitment of death domain DD containing adaptors such as Fas  [ **FADD** ]  associated death domain  **FADD**  and TNFR  [ **TNFRSF1A** ]  associated DD TRADD can lead to the activation of a signal transduction pathway that induces apoptosis, recruitment of TRAF family proteins can lead to the activation of transcription factors such as, NF kappaB and JNK thereby promoting cell survival and differentiation as well as immune and inflammatory responses.  Ref: 12787559 Cytokine Growth Factor Rev, 2003
- The best studied death inducing ligand receptor pairs are TNF TNF receptor 1 TNFR1  [ **TNFRSF1A** ]  and CD95L CD95 Fas  [ **FADD** ] , Apo 1.  Ref: 12628743 Toxicol Lett, 2003
- By contrast, in neurons, reovirus induced apoptosis involves a wider array of DRs, including TNFR  [ **TNFRSF1A** ]  and Fas  [ **FADD** ] , and the mitochondria appear to play a less critical role.  Ref: 12766474 Apoptosis, 2003
- **FADD**  and caspase 8 are not recruited to the TNF R1  [ **TNFRSF1A** ]  signaling complex during TNF induced apoptosis.  Ref: 12721308 J Biol Chem< MedlineTA>J Biol Chem, 2003
- The constitutive expression of mRNA for TNF receptors TNFR  [ **TNFRSF1A** ] , including TNFRI and TNFRII, and the adapter molecules, such as the TNF receptor associated death domain protein TRADD, Fas  [ **FADD** ]  associated death domain protein  **FADD** , receptor interacting protein RIP and TNF receptor associated factor 2 TRAF 2 were analyzed by reverse transcriptase RT PCR in bone marrow samples from control, MDS and AML cases.  Ref: 12681357 Leuk Res, 2003
- The induction of IkappaBalpha degradation by DNA damage was normal in TNF R1  [ **TNFRSF1A** ] , TRAF2, TRAF5 and  **FADD**  fibroblasts or when de novo protein synthesis was blocked.  Ref: 12654725 Genes Dev, 2003
